# Supplementary material for: Purkinje Cell Activity Resonation Generates Rhythmic Behaviors at the Preferred Frequency of 8 Hz
Source: Biomedicines. 2022 Jul 29;10(8):1831. doi: 10.3390/biomedicines10081831 (PMC9404806; doi:10.3390/biomedicines10081831)
Supplement: Supplementary file 1 [file biomedicines-10-01831-s001.zip › biomedicines-1818771-supplementary.pdf]

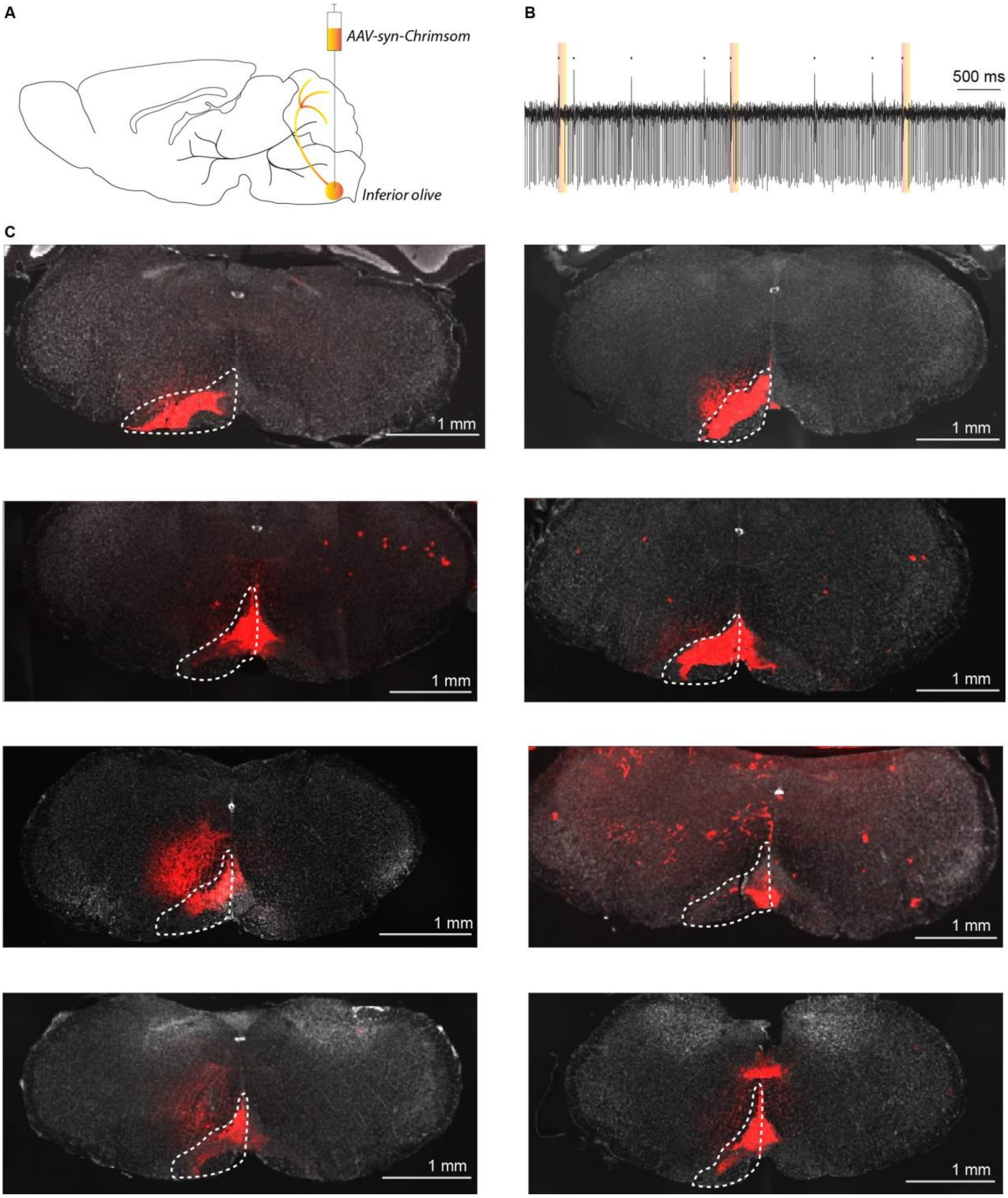

Figure S1. Histological verification of the viral injections in the inferior olive. (**A**) Schematic of the viral injection targeting the inferior olive. (**B**) Representative Purkinje cell recording in which complex spikes were elicited by climbing fiber stimulation at 0.5 Hz in the right cerebellar hemisphere. (**C**) Coronal sections the brainstem of 8 mice that received a Chrimson virus injection in the left inferior olive. The labelling represents the expression of fluorescent td-tomato protein which was used to verify the site of the injection.

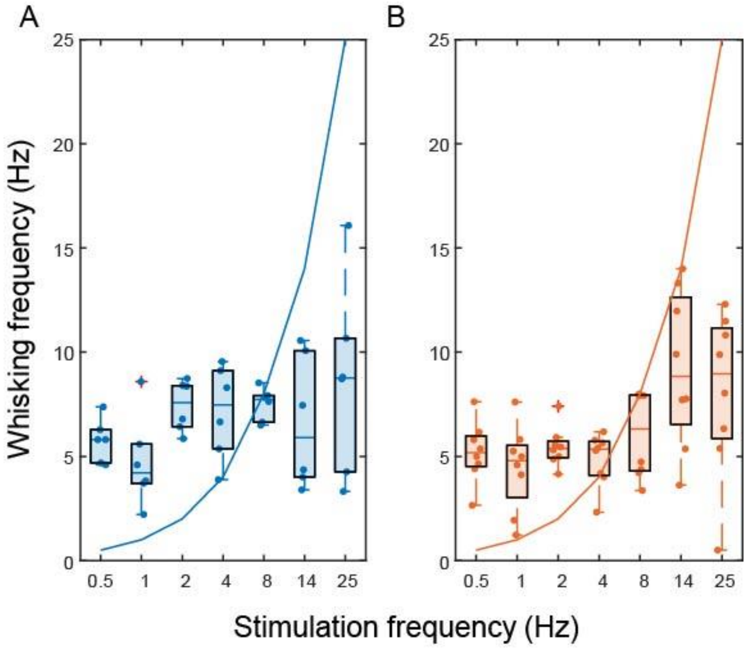

Figure S2. Instantaneous frequency of whisker movements during optogenetic stimulation. In addition to extracting the peak of the power spectrum (Fig. 3), we calculated the instantaneous frequency of whisker movements over time and then we calculated the mean of all components. (**A**) Boxplot of the mean instantaneous whisker frequency during simple spike stimulation. Dots represent individual mice and crosses the outliers (n= 6). The blue line indicates the frequency of stimulation. At 8 Hz mice whisk at the same frequency of stimulation. (**B**) Same as (**A**), but for climbing fiber stimulation.
